# CD8^+^ Tregs ameliorate inflammatory reactions in a murine model of allergic rhinitis

**DOI:** 10.1186/s13223-021-00577-8

**Published:** 2021-07-22

**Authors:** Lin Lin, Fei Dai, Jinjin Wei, Zheng Chen

**Affiliations:** Department of Otorhinolaryngology-Head and Neck Surgery, Huashan Hospital of Fudan University, No. 12 Wulumuqi Middle Road, Shanghai, 200040 China

**Keywords:** CD8^+^CD25^+^ Foxp3^+^ regulatory T cells, Allergic rhinitis, Mouse, Model, Nasal mucosa

## Abstract

**Background:**

CD8^+^CD25^+^fork-head box transcription factor (Foxp3)^+^ regulatory T cells (CD8^+^ Tregs) play a role in immune tolerance. However, the role of these cells in allergic rhinitis (AR) has not been elucidated. The study aimed to evaluate influences of CD8^+^ Tregs on inflammatory conditions in a murine model of AR.

**Methods:**

A murine model of AR was established. CD8^+^ Tregs were isolated from mice nasal mucosa and cultured in vitro. We examined interleukin (IL)-10 and transforming growth factor (TGF)-β in cell cultures. Then, we administered CD8^+^ Tregs into mice nasal mucosal cultures, and examined eosinophil cation protein (ECP), IL-4, IL-5 and IL-13 in these cultures. Finally, we adoptively transferred CD8^+^ Tregs into mice models, and evaluated percentages of CD8^+^ Tregs, numbers of sneezing and nasal rubbing, and counts of eosinophils and contents of ECP, IL-4, IL-5, IL-13, IL-10 and TGF-β in nasal lavage fluid (NLF) in mice.

**Results:**

The percentage of CD8^+^ Tregs from AR mice was reduced. IL-10 and TGF-β were increased in cell cultures from AR mice. ECP, IL-4, IL-5 and IL-13 were decreased after the AR mice CD8^+^ Tregs administration in mucosal cultures. However, their contents were not changed after normal CD8^+^ Tregs treatment. Additionally, the adoptive transfer of AR CD8^+^ Tregs enhanced the percentage of CD8^+^ Tregs and levels of IL-10 and TGF-β in NLF, reduced numbers of sneezing and nasal rubbing, and counts of eosinophils and concentrations of ECP, IL-4, IL-5 and IL-13 in NLF. However, normal CD8^+^ Tregs could not change above parameters.

**Conclusion:**

These findings show that CD8^+^ Tregs may inhibit inflammatory responses in the AR condition.

## Background

Allergic rhinitis (AR) is an immunoglobulin (Ig) E-mediated inflammatory disease. This condition has a significant influence on patient morbidity, and is a major economic burden to society [1]. The prevalence of self-reported AR has been estimated to be approximately 1 to 40% in adults around the whole globe [2, 3]. A similar survey over a 6-year period in the general Chinese adult population reports that the standardized prevalence of adult AR increases from 11.1% in 2005 to 17.6% in 2011 in China mainland [4].

AR is characterized as specific IgE-mediated reactions against inhaled allergens driven by type 2 helper T (Th2) cells. This chronic condition results in nasal mucosal inflammation involving multiple cell types, such as dendritic cells, B cells, mast cells, eosinophils and basophils [5, 6], and various cytokines, such as interleukin (IL)-4, IL-5, IL-9 and IL-13 [7]. Th2 cells play a significant role in the initiation, progression and persistence of allergic diseases including AR and asthma. Immunoregulatory mechanisms, which may regulate the severity, persistence or alleviation of this chronic disease, are also investigated further.

In recent years, different regulatory T cells (Tregs) subsets have been identified, such as CD4^+^CD25^+^fork-head box transcription factor (Foxp3)^+^ Tregs (CD4^+^ Tregs), Tregs type-1 cells, Th3 and CD8^+^CD25^+^Foxp3^+^ Tregs (CD8^+^ Tregs) [8]. CD4^+^ Tregs have been reported to reverse aberrant Th2-mediated allergic responses including AR and asthma [9]. However, the potential role of CD8^+^ Tregs in the pathophysiological mechanisms of AR has not been fully elucidated. This phenotype of Tregs has been identified successfully in human and murine studies only in recent years [10–12]. One of these studies demonstrates that CD8^+^ Tregs are associated with the tumor progression in human gastric cancer [11]. Another study indicates that T cell receptor stimulation with staphylococcus enterotoxin B expands CD8^+^ T cell population and induces CD8^+^ Tregs [12]. These investigations suggest that CD8^+^ Tregs may display suppressive activities in tumor-induced immunosuppressive networks and autoimmune diseases. However, little is known about the mechanisms of whether CD8^+^ Tregs inhibit the inflammation in allergic conditions, which our research groups have been investigating.

CD4^+^ Tregs regulate the Th1/Th2 immune balance and reduce the activity of Th1 cells in AR mice [13]. As for CD8^+^ Tregs, their cellular activities in AR have not been fully understood. Based on previous researches, we hypothesized that these Tregs might contribute to the suppression of inflammatory reactions in AR. And we performed this study to explore the influences of CD8^+^ Tregs on inflammatory conditions in a murine model of AR.

## Methods

### Mice

Female BALB/c mice were 6–8 weeks of age and purchased from the Chinese Academy of Sciences Shanghai Laboratory Animal Center. The mice were raised in horizontal laminar flow cabinets and provided sterile food and water in a specific pathogen-free facility. This animal study was approved by the Institutional Animal Care and Use Committee of Fudan University (Ethic No. 2019 Huashan Hospital JS-071). These animals were randomly divided into four groups (n = 6 for each group).

### AR models

**Fig. 1:**
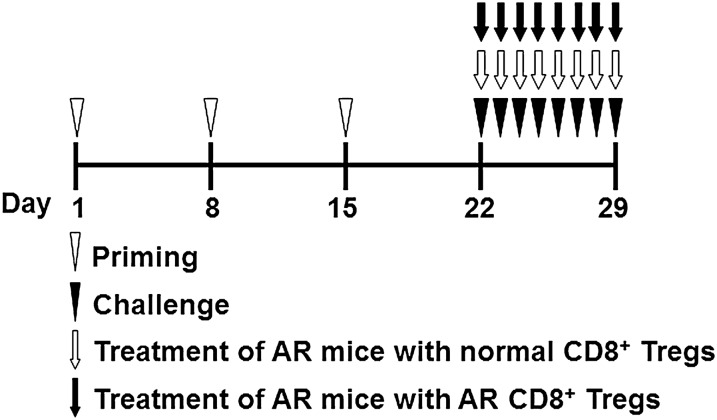
Study protocols. A murine model of allergic rhinitis (AR) was prepared by intraperitoneal immunization of ovalbumin on days 1, 8, and 15 (priming) followed by daily intranasal ovalbumin challenge on days 22 to 29. This group of mice was used as allergic models (AR group). As a negative control, another group of mice received the challenge treatment of normal saline alone (normal group). A group of allergic mice received the adoptive transfer of CD8^+^CD25^+^fork-head box transcription factor (Foxp3)^+^ regulatory T cells (CD8^+^ Tregs) from normal mice (normal CD8^+^ Tregs group) or allergic mice (AR CD8^+^ Tregs group) intravenously in the tail vein on challenging days

According to the published protocols [14], a group of mice were administered 0.5 mg/mL of ovalbumin (OVA, grade V; Sigma-Aldrich, St. Louis, Missouri, USA) and 20 mg/mL of aluminium hydroxide (Sinopharm Chemical Reagent Co Ltd., Shanghai, China) in normal saline at a dosage of 0.2 mL/mouse through the intraperitoneal injection. The sensitization process was repeated three times at weekly intervals (days 1, 8 and 15). After that, these mice were challenged by the daily instillation of OVA solution droplet (40 mg/mL in normal saline) into the nostrils (0.02 mL/mouse) with a micropipette on days 22 to 29 (Fig. 1). This group of mice was used as allergic models (AR group). As a negative control, another group of mice received the challenge treatment of normal saline alone (normal group). A group of allergic mice received the adoptive transfer of CD8^+^ Tregs from normal mice (normal CD8^+^ Tregs group) or allergic mice (AR CD8^+^ Tregs group) cultured in vitro intravenously in the tail vein on challenging days. Nasal symptoms were assessed by counting numbers of sneezing and nasal rubbing during 10 min immediately after the last OVA intranasal provocation on day 29.

### Samples preparation

After mice were sacrificed, the fore teeth were cut off. The lower jaw and cheek muscles were removed. Nasal mucosa samples were collected and cut into two portions: One was for flow cytometry analysis, and the other was cultured in vitro.

### Flow cytometry analysis

Nasal mucosa samples were processed into cell suspensions. Then the cell suspensions were centrifuged for 10 min at 200×*g* at 4 °C, and cells were harvested for flow cytometry analysis. CD8^+^ cells were isolated using mouse CD8 microbeads according to the manufacturer’s protocol (Miltenyi Biotec, Bergisch Gladbach, Germany). CD8^+^CD25^+^ T cells were separated using an EasySep™ mouse CD8^+^ T cells enrichment kit (StemCell Technologies, Vancouver, BC, Canada), followed by isolation with CD25 microbeads (Miltenyi Biotec, Bergisch Gladbach, Germany). For intracellular staining, these cells were stained with Foxp3 staining kit (MyBioSource, Inc., San Diego, CA, USA). Finally, above cells were resuspended and analyzed using a FACSAria flow cytometer (BD Biosciences, San Jose, CA, USA) and FlowJoSoftware (TreeStar Inc., Ashland, OR, USA).

### Cell cultures

CD8^+^ Tregs were cultured in vitro at 5 × 10^5^/well in 96-well plates coated with anti-CD3. IL-2 was then added at 1000 IU/mL. The cell cultures were kept for three days. The cell supernatants were used to measure levels of cytokines IL-10 and transforming growth factor (TGF)-β with enzyme-linked immunosorbent assay (ELISA), and the cell lysates were used to assess the contents of messenger (m) RNAs of IL-10 and TGF-β by using real-time reverse transcription–polymerase chain reaction (RT-PCR). After that, CD8^+^ Tregs were resuspended in normal saline, and were adoptively transferred at 100 µL per mouse (5 × 10^5^ cells/mouse) intravenously in the tail vein on challenging days.

### Nasal lavage fluid (NLF) preparation

After mice were killed, one blunted 18-gauge needle was used to obtain NLF. One injection of 2000 μL normal saline was performed and the fluid was collected with a tube under both nares of the mouse nose for NLF [15]. A portion of NLF was centrifuged for 10 min at 150×*g* at 4 °C. The supernatants were stored at − 70 °C for eosinophil cation protein (ECP), IL-4, IL-5, IL-13, IL-10 and TGF-β assays. Another portion was collected for detection of eosinophils. Differential cell counts on 150 cells were performed on cytospins (Cytospin 4 Shandon Ltd., Runcorn, UK) stained with Giemsa [16].

### Nasal mucosal cultures

Nasal mucosa from normal and AR mice were cultured in vitro in accordance with published protocols [17]. To assess the impact of CD8^+^ Tregs treatment on allergic condition, nasal mucosa from AR mice were saturated for 1 h in culture medium with DMEM and 10% calf serum and 10 μg/mL gentamicin in the presence of CD8^+^ Tregs (5 × 10^5^) from normal or AR mice cultured in vitro for three days, and then placed on a hydrated 1 × 1 cm gelatin sponge with the submucosa downward and the mucosa facing upward. Nasal mucosa from normal mice were not treated using CD8^+^ Tregs. Finally, all the cultured samples were collected and stored at –20 °C for further examinations. The tissue culture supernatants were used to measure mediator concentrations of ECP, IL-4, IL-5 and IL-13.

### ELISA

IL-10 and TGF-β in cell cultures, ECP, IL-4, IL-5 and IL-13 in tissue cultures, and all these mediators in NLF were evaluated using corresponding ELISA kits which were all purchased from MyBioSource, Inc., San Diego, CA, USA. The ELISA procedures were strictly performed according to the manufacturers' protocols.

### Real-time RT-PCR

Real-time RT-PCR was performed to evaluate the mRNA of IL-10 and TGF-β in CD8^+^ Tregs cultures. Briefly, the total RNA was extracted with Trizol (Invitrogen, Carlsbad, CA, USA) and treated with RNase-free DNase. For reverse transcription, 2 μg of the above RNA was reversely transcribed with random hexamers (Invitrogen, Carlsbad, CA, USA) and cDNA was amplified in accordance with the manufacturer’s instructions. IL-10 mRNA primers were as follows: forward primer 5′-TCCATCATG CCTGGCTCA-3′, and reverse primer 5′-GGTGTTTTAGCTTTTCATTTT-3′. TGF-β mRNA primers were as follows: forward primer 5′-ATTCCTGGCGTTACCTTGG-3′, and reverse primer 5′-AGCCCTGTATTCCGTCTCCT-3′. GAPDH mRNA primers were as follows: forward primer 5′-ACCACAGTCCATGCCATCAC-3′ and reverse primer 5′-TCCACCACCCTGTTGCTGTA-3′. After initial denaturation at 95 °C for 10 min, the amplification profile was 15 s of denaturation at 95 °C, 1 min of annealing and extension at 60 °C for 45 cycles. For the measurement 2 μL of diluted cDNA was amplified in a total reaction volume of 20 μL by using an 7500 real-time PCR System (Applied Biosystems, Foster City, CA, USA) with 20 × SYBR Green mixture (Invitrogen, Carlsbad, CA, USA). Evaluation of data was performed using the ΔCT method with GAPDH as the internal standard.

### Statistical analysis

Statistical analysis was performed by using a commercially available statistical software prism 6.0 (GraphPad Software Inc., San Diego, California, USA). Student’s *t* test was used for comparisons between groups and data are presented as mean ± SEM (standard error of the mean). A *p*-value of less than 0.05 was considered statistically significant.

## Results

### CD8^+^ Tregs in AR mice

**Fig. 2:**
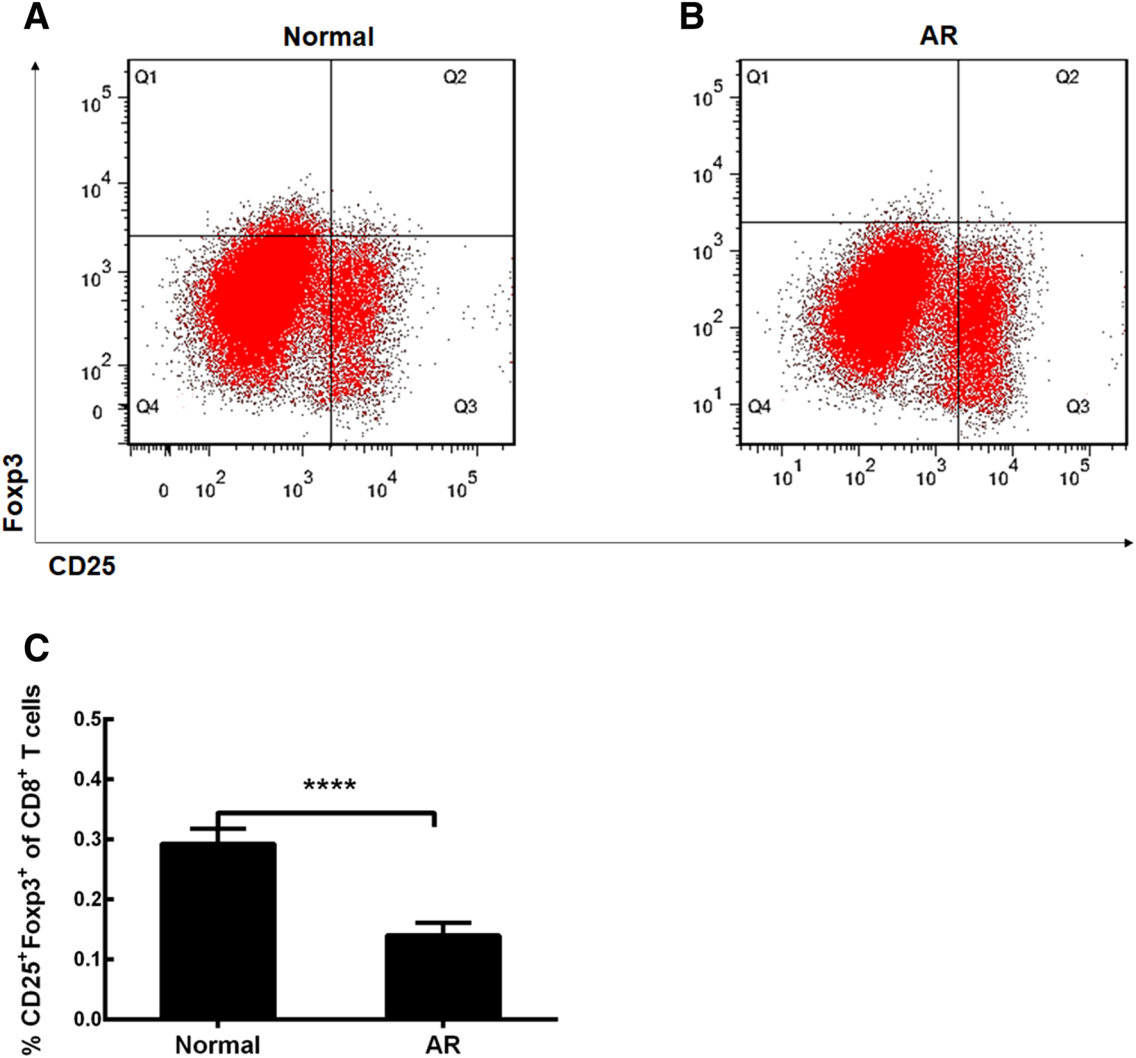
Flow cytometry analysis of CD8^+^CD25^+^fork-head box transcription factor (Foxp3)^+^ regulatory T cells (CD8^+^ Tregs). **A** CD8^+^ Tregs in normal mice. **B** CD8^+^ Tregs in allergic mice. C, Comparisons of percentage of CD8^+^ Tregs in total CD8^+^ T cells. Normal, normal mice; AR, allergic rhinitis mice. The values shown are expressed as mean ± SEM. ****p < 0.0001

We made this relevant experiment to detect the percentage of these Tregs from normal and allergic mucosa in mice using flow cytometry. This type of Tregs was identified as the CD8^+^CD25^+^Foxp3^+^ cell population (Fig. 2A, B). We found that the CD8^+^ Tregs percentage in total CD8^+^ T cells in AR mice was decreased compared with that in normal mice (Fig. 2 C, *p* < 0.0001, AR group vs Normal group). In this study, we used Tregs from nasal mucosa rather than peripheral blood mononuclear cells, just because these local cells were closer to their real allergic environment in AR. The obtained data show that the decrease of Tregs may play a role in the initiation and development of AR.

### IL-10 and TGF-β in CD8^+^ Tregs cultures

**Fig. 3:**
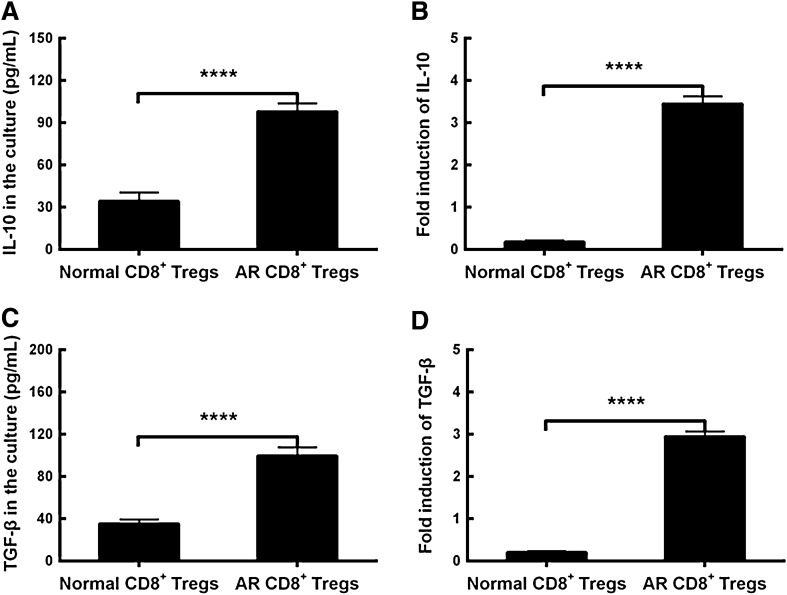
Comparisons of interleukin (IL)-10 and transforming growth factor (TGF)-β in CD8^+^CD25^+^fork-head box transcription factor (Foxp3)^+^ regulatory T cells (CD8^+^ Tregs) cultures. **A** Comparisons of IL-10 between Normal and AR CD8^+^ Tregs. **B** Comparisons of IL-10 mRNA between Normal and AR CD8^+^ Tregs. **C** Comparisons of TGF-β between Normal and AR CD8^+^ Tregs. **D** Comparisons of TGF-β mRNA between Normal and AR CD8^+^ Tregs. The values shown are expressed as mean ± SEM. ****p < 0.0001

To make a further understanding of the activity of CD8^+^ Tregs, we examined the inhibitory cytokines IL-10 and TGF-β in the cell cultures. The data demonstrated that the expressing levels of IL-10 and TGF-β were upregulated in cell cultures from allergic mucosal tissues compared to those from normal mucosa (Fig. 3A, C, p < 0.0001, AR CD8^+^ Tregs vs Normal CD8^+^ Tregs). As for the mRNAs of IL-10 and TGF-β, the contents were increased in CD8^+^ Tregs from AR mice when compared to those from normal mice (Fig. 3B, D, p < 0.0001, AR CD8^+^ Tregs vs Normal CD8^+^ Tregs). The findings show that the elevated production and release of IL-10 and TGF-β from this type of Tregs may imply their cellular activation state in the inflammatory state of AR. On the contrary, CD8^+^ Tregs may in relatively static status when they are in normal mucosa.

### Inflammatory mediators in mucosal cultures

**Fig. 4:**
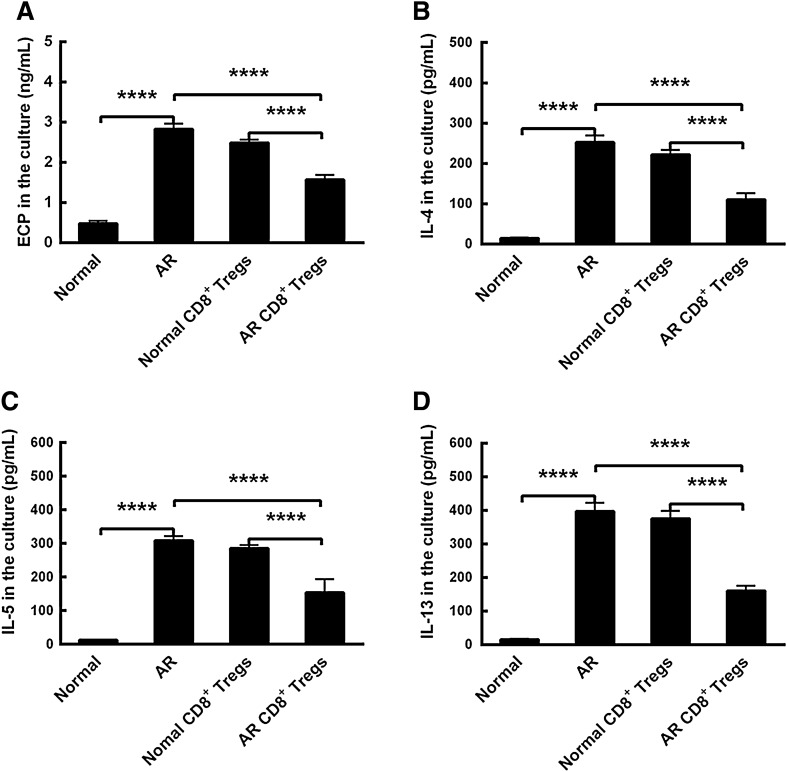
Comparisons of inflammatory mediators in nasal mucosal cultures. **A** eosinophil cation protein (ECP) in the culture. **B** interleukin (IL)-4 in the culture. **C** IL-5 in the culture. **D** IL-13 in the culture. Normal, normal mucosa. AR, allergic rhinitis mucosa; Normal CD8^+^ Tregs, normal CD8^+^ Tregs-treated allergic rhinitis mucosa; AR CD8^+^ Tregs, AR CD8^+^ Tregs-treated allergic rhinitis mucosa. The values shown are expressed as mean ± SEM. ****p < 0.0001

In order to investigate the cellular functions of CD8^+^ Tregs, we administered these Tregs and evaluated the type-2 inflammatory mediators profile including ECP, IL-4, IL-5 and IL-13 in mucosal tissue cultures. The results displayed that all these mediators were increased in AR mucosa cultures compared to those in normal mucosa ones (Fig. 4A–D, p < 0.0001, AR vs Normal). After normal CD8^+^ Tregs administration, these mediators were not changed statistically (Fig. 4A–D, Normal CD8^+^ Tregs vs AR). However, AR CD8^+^ Tregs administration decreased their expressing levels significantly in vitro (Fig. 4A–D, p < 0.0001, AR CD8^+^ Tregs vs AR). Furthermore, there were significant differences in contents of these type-2 mediators between these two treatments (Fig. 4A–D, p < 0.0001, AR CD8^+^ Tregs vs Normal CD8^+^ Tregs). The findings suggest that CD8^+^ Tregs-conditioned media may reduce the allergic inflammation in vitro.

### CD8^+^ Tregs in AR mice after the adoptive transfer of cultured CD8^+^ Tregs

**Fig. 5:**
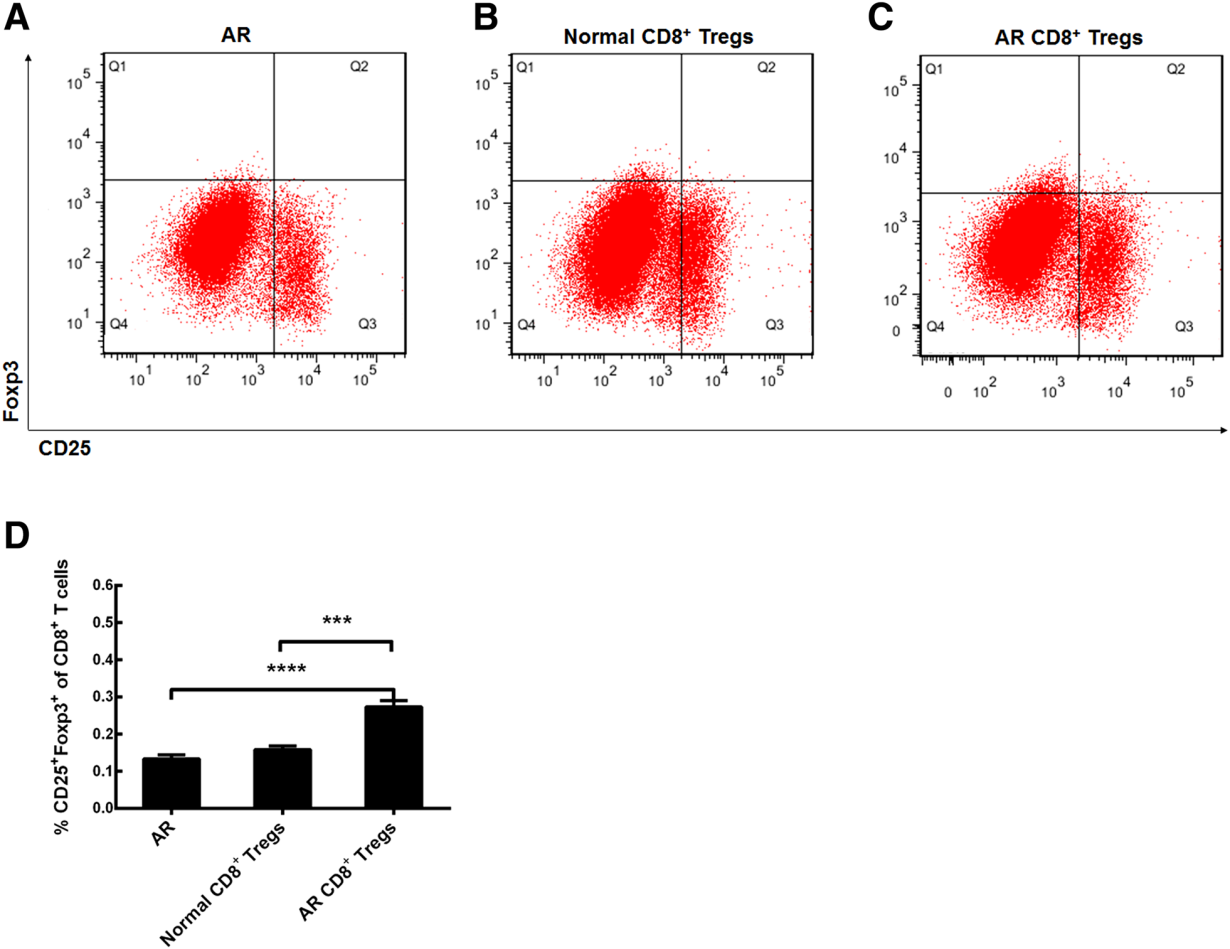
CD8^+^ Tregs in AR mice after the adoptive transfer of cultured CD8^+^ Tregs in vitro. **A** CD8^+^ Tregs in allergic mice. **B** CD8^+^ Tregs in allergic mice after the normal CD8^+^ Tregs treatment. **C** CD8^+^ Tregs in allergic mice after the AR CD8^+^ Tregs treatment. **D** Comparisons of percentages of CD8^+^ Tregs in allergic mice. AR, allergic rhinitis group; Normal CD8^+^ Tregs, treatment of AR mice with normal CD8^+^ Tregs; AR CD8^+^ Tregs, treatment of AR mice with AR CD8^+^ Tregs. The values shown are expressed as mean ± SEM. ***p < 0.001. ****p < 0.0001

To explore the trafficking of cultured CD8^+^ Tregs in vitro into mice nasal mucosa, we examined percentages of CD8^+^ Tregs in AR mice mucosa after the adoptive transfer of normal or AR CD8^+^ Tregs through the tail vein using flow cytometric analysis (Fig. 5A–C). The data indicated that there were no statistical differences between AR group and normal CD8^+^ Tregs-treated group (Fig. 5D). However, the CD8^+^ Tregs percentage in total CD8^+^ T cells from nasal mucosa was enhanced after the AR CD8^+^ Tregs treatment (Fig. 5D, p < 0.0001, AR CD8^+^ Tregs vs AR). Not surprisingly, the percentage of these Tregs in AR CD8^+^ Tregs treatment group was also greater than that in normal CD8^+^ Tregs treatment one (Fig. 5D, p < 0.001, AR CD8^+^ Tregs vs Normal CD8^+^ Tregs). The results show that allergic CD8^+^ Tregs are likely to traffic into allergic nasal mucosa of mice from their tail veins through some unknown mechanisms.

### Alleviation of the allergic inflammation by AR CD8^+^ Tregs in vivo

**Fig. 6:**
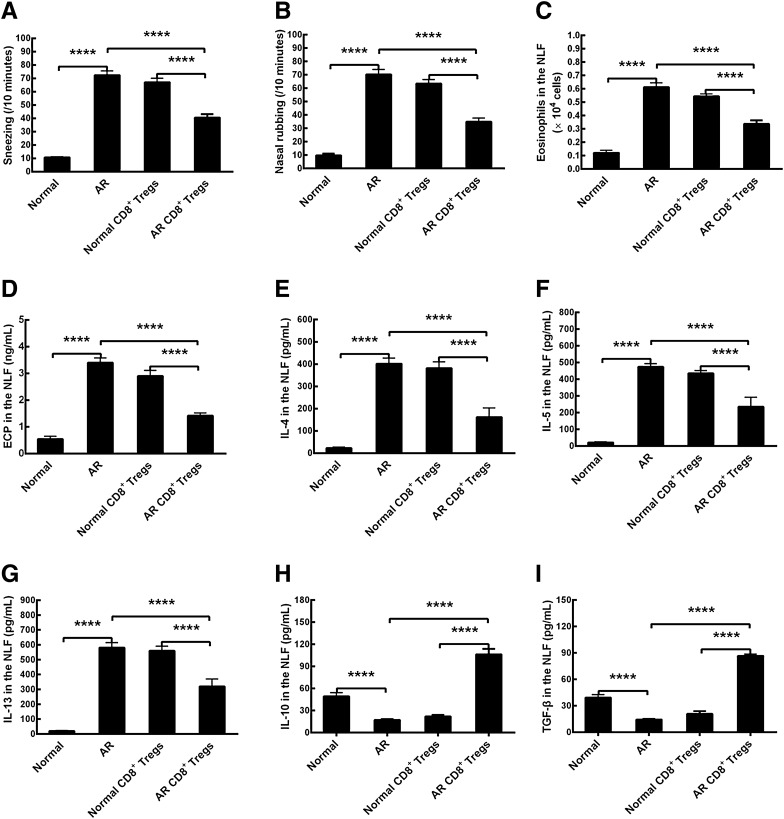
Alleviation of allergic inflammation by the adoptive transfer of CD8^+^CD25^+^fork-head box transcription factor (Foxp3)^+^ regulatory T cells (CD8^+^ Tregs) in a murine model of allergic rhinitis (AR). **A** Number of sneezing. **B** Number of nasal rubbing. **C** Number of eosinophils in the nasal lavage fluid (NLF). **D** eosinophil cation protein (ECP) in the NLF. **E** interleukin (IL)-4 in the NLF. **F** IL-5 in the NLF. **G** IL-13 in the NLF. **H** IL-10 in the NLF. **I** Transforming growth factor (TGF)-β in the NLF. Normal, normal group; AR, allergic rhinitis group; Normal CD8^+^ Tregs, treatment of AR mice with normal CD8^+^ Tregs; AR CD8^+^ Tregs, treatment of AR mice with AR CD8^+^ Tregs. The values shown are expressed as mean ± SEM. ****p < 0.0001

To evaluate the effects of OVA-induced allergic responses, sneezing and nasal rubbing were counted after the last allergen challenge. Allergic mice (AR group) showed enhancements in counts of sneezing and nasal rubbing compared to those of normal mice (Normal group) that had received only normal saline challenge (Fig. 6A and B, p < 0.0001, AR vs Normal). The number of eosinophils in the NLF of AR mice indicated an increase compared to that in normal mice (Fig. 6C, p < 0.0001, AR vs Normal). Similarly, type-2 inflammatory mediators including ECP, IL-4, IL-5 and IL-13 in NLF were also upregulated in allergic mice in comparison with those in normal mice (Fig. 6D–G, p < 0.0001, AR vs Normal). The results clearly demonstrated that the murine model of AR was established successfully. The adoptive transfer of CD8^+^ Tregs obtained from normal mice into AR mice did not reduce numbers of sneezing and nasal rubbing, and eosinophil counts and concentrations of ECP, IL-4, IL-5, and IL-13 in NLF when compared with those in AR control (Fig. 6A–G, Normal CD8^+^ Tregs vs AR). However, the cultured CD8^+^ Tregs from AR mice models administration lessened sneezing and nasal rubbing numbers, eosinophil infiltrates and levels of above mediators (Fig. 6A–G, p < 0.0001, AR CD8^+^ Tregs vs AR). Additionally, there were statistical differences in the treatment of allergic mice using AR and normal CD8^+^ Tregs in all the above parameters (Fig. 6A–G, p < 0.0001, AR CD8^+^ Tregs vs normal CD8^+^ Tregs). In other words, the adoptive transfer of AR CD8^+^ Tregs did reduce the inflammatory reactions in AR condition. As for IL-10 and TGF-β, their concentrations in the NLF of mice were decreased in AR mice (Fig. 6H, I, p < 0.0001, AR vs Normal), and were increased after the transfer of CD8^+^ Tregs from AR mice (Fig. 6H, I, p < 0.0001, AR CD8^+^ Tregs vs AR). However, these two inhibitory cytokines were not changed statistically after the normal CD8^+^ Tregs administration (Fig. 6H, I). We also found that there were statistical differences in their contents in the NLF between these two treatments (Fig. 6H, I, p < 0.0001, AR CD8^+^ Tregs vs Normal CD8^+^ Tregs). The data illustrate that CD8^+^ Tregs are activated in allergic state and perform their functions in the suppression of local inflammation in allergic diseases, just as CD4^+^ Tregs do.

## Discussion

AR is defined as a chronic disease with symptoms of sneezing, nasal pruritus, nasal obstruction and nasal discharge. Pharmacologic therapies are always targeted mediators, cytokines, or nonspecific inflammation in order to improve allergic symptoms [18]. Although these medications reduce the allergic inflammation, the specific allergen immunotherapy (subcutaneous or sublingual allergen immunotherapy) is so far the only potential curative therapy for AR. Immunotherapy can change Th2-driven responses through various mechanisms, such as elevated IgG4 blocking antibody and increased CD4^+^ Tregs that can produce immunomodulatory cytokines like IL-10 and TGF-β [19]. As for the effects of immunotherapy on CD8^+^ Tregs, we have not found relevant studies. Generally, the potential role of CD8^+^ Tregs on the allergic inflammation is not involved up to now whether in clinical trials or in animal experiments.

CD8^+^ Tregs were first observed in the early 1970s [20]. Nonetheless, these Tregs have been clearly identified until recently. CD8^+^ Tregs were first described as a subset of single positive CD8^+^ thymocytes sharing phenotypic, functional and mechanistic features with CD4^+^ Tregs [21]. Later, several researches show that some CD8^+^ Tregs can suppress immunity non-specifically [22, 23], whereas others are antigen-specific, such as viral, allogeneic and self-antigen [24–26]. An interesting study demonstrates that CD8^+^ Tregs have the capacity in suppressing prostate cancer mainly through a mechanism that is dependent on cell contact [27]. Lately, some scholars have found that CD8^+^ Tregs were reduced in chronic rhinosinusitis, and they conclude that the decrease of these Tregs indicates an inflammatory bias and the inability to regulate chronic mucosal disease [28]. Unfortunately, the authors do not go a further step to explore the exact roles of CD8^+^ Tregs in the pathophysiological mechanisms of chronic rhinosinusitis.

In this present study, we aimed to evaluate the cellular functions of CD8^+^ Tregs in allergic condition of AR mice models. Firstly, we established a murine model of AR by using OVA and its adjuvant. Then, CD8^+^ Tregs were isolated and purified from mice normal and allergic mucosa. We found the percentage of CD8^+^ Tregs in total CD8^+^ T cells in AR mice was decreased significantly compared with that in normal mice, which might suggest their possible suppressive capacity at the mucosal site through cell–cell contacts or other ways in the allergic inflammation. The data might not be novel, but might confirm what was previously suggested or demonstrated particularly in the AR condition [29]. Tregs deficiencies might not be the primary cause of allergy as previously suggested [30]. However, a deficiency of Tregs would be presented much later on in adulthood if AR patients had not been exposed to allergens much earlier on in childhood [30].

To explore the cellular activities of the CD8^+^ Tregs, we determined the contents of IL-10 and TGF-β and their corresponding mRNAs in the cell cultures in vitro. Our data showed that expressing levels of these suppressive cytokines and their mRNAs were all upregulated in cell cultures from allergic mucosal tissues compared to those from normal mucosa. The results revealed that CD8^+^ Tregs might be in the activating state in the allergic condition and be in the static state in normal mucosa. The pathological mechanisms about how these Tregs were differentiated and activated in AR were required to be further elucidated.

To investigate the cellular functions of these Tregs, we applied CD8^+^ Tregs cultured in vitro into allergic mucosal cultures and examined the type-2 inflammatory mediators profile including ECP, IL-4, IL-5 and IL-13. We found all the mediators were increased in AR mucosa cultures compared to those in normal mucosa ones. After the normal CD8^+^ Tregs treatment, these mediators were not lowered statistically. However, the AR CD8^+^ Tregs administration decreased their concentrations significantly. Not surprisingly, there were statistical differences between these two treatments in contents of these substances. The findings clearly told us that CD8^+^ Tregs could alleviate the allergic inflammation in vitro.

To further make a better understanding of the suppressive functions of CD8^+^ Tregs, we performed an in vivo study in a murine model of AR. First of all, we explored the migration of cultured CD8^+^ Tregs in vitro in mice with flow cytometry. The obtained data demonstrated that the total CD8^+^ Tregs percentage in nasal mucosa of AR mice models was not changed after the adoptive transfer of CD8^+^ Tregs from normal mice, and was elevated significantly after the allergic CD8^+^ Tregs treatment. The results imply that the activated CD8^+^ Tregs may migrate into the nasal mucosa of mice under allergic circumstances from the tail vein to the blood circulation and to the local mucosa by some mechanisms which are required to be explained.

After the establishment of AR models, we found counts of sneezing and nasal rubbing, and eosinophils and type-2 inflammatory mediators including ECP, IL-4, IL-5 and IL-13 in NLF were all enhanced in AR group compared to those in normal group. However, the levels of IL-10 and TGF-β were decreased in AR mice, which characterized the type-2 allergic inflammation. The adoptive transfer of CD8^+^ Tregs acquired from normal mice into AR mice did not reduce above parameters. The CD8^+^ Tregs from AR mice treatment decreased sneezing and nasal rubbing numbers, eosinophil invasions and concentrations of the above proinflammatory mediators, and upregulated anti-inflammatory cytokines including IL-10 and TGF-β in mice NLF. In brief, the adoptive transfer of AR CD8^+^ Tregs did ameliorate the inflammatory responses in AR mice. These data illuminated that the local CD8^+^ Tregs were activated in the allergic state and fulfilled their functions in suppressing the nasal allergic inflammation, just as the conventional CD4^+^ Tregs did. CD8^+^ Tregs from allergic mice were so potent in ameliorating the allergic inflammation, why were they unable to control the inflammation in the mice from which they were obtained? The reason might be that this Treg population was very small and their cellular activities were restrained by currently unknown mechanisms. In addition, we did not investigate the way by which CD8^+^ Tregs trafficked to the nasal mucosa after the adoptive transfer through the tail vein, and the precise way by which these Tregs downregulated the inflammation locally and/or systemically. Briefly, the exact mechanisms of inhibitory activities of these Tregs in the AR disease are required to be completely investigated.

It is well known that CD8^+^ and CD4^+^ Tregs recognize their cognate antigens presented by major histocompatibility complex class I molecule (MHC-I) or MHC-II on relevant cell types, respectively. Hence, CD8^+^ Tregs will display their suppressive activities on virtually all cells, whereas CD4^+^ Tregs’ suppressive abilities will occur only on cells expressing MHC-II molecules [31]. That is to say, CD8^+^ Tregs might reduce the allergic inflammation more than CD4^+^ Tregs do, whether in the innate or the adaptive immune system. Based on these, we think it very important to investigate the functional mechanisms of CD8^+^ Tregs and to exploit the CD8^+^ Treg-cell therapy. Cell therapy has developed greatly in recent years in the field of autoimmune diseases and transplantation with promising results. Therefore, the CD8^+^ Treg-cell therapy might be a promising treatment method in allergic diseases including AR and asthma.

## Conclusion

In conclusion, the results of this study show that CD8^+^ Tregs may play an inhibitory role in inflammatory reactions in the AR condition. It should be emphasized that CD8^+^ Tregs might offer a novel and effective therapeutic and/or prophylactic means to treat AR in the future. Additionally, there are some issues required to be considered, such as factors resulting in the diminished CD8^+^ Tregs in the allergic condition, the endogenous increase of CD8^+^ Tregs in nasal mucosa by the pharmacologic intervention, and the influence of immunotherapy in AR patients on CD8^+^ Tregs [32]. Furthermore, it is well-known that epigenetic mechanisms play a pivotal role in the regulation of T cells function [33] and also in CD8^+^ cells [34]. Consequently, relevant studies are required to be performed to investigate the relationships between epigenetics and CD8^+^ Tregs.

## Data Availability

The datasets used and analyzed during the current study are available from the corresponding author on reasonable request.
